# Efficacy and safety of CO_2_ cryotherapy in the treatment of infants with tracheobronchial tuberculosis

**DOI:** 10.3389/fped.2022.984738

**Published:** 2022-10-10

**Authors:** Yidi Zhao, Tongqiang Zhang, Nan Yang, Yongsheng Xu, Wei Guo

**Affiliations:** ^1^Children's Clinical College of Tianjin Medical University, Tianjin Children's Hospital/Tianjin University Children's Hospital, Tianjin, China; ^2^Department of Pulmonology, Tianjin Children's Hospital/Tianjin University Children's Hospital, Tianjin, China; ^3^Department of Imaging, Tianjin Children's Hospital/Tianjin University Children's Hospital, Tianjin, China

**Keywords:** tracheobronchial tuberculosis, CO_2_ cryotherapy, bronchoscopy, lymph node fistula, infants

## Abstract

**Objective:**

This study aimed to investigate the efficacy and safety of CO_2_ cryotherapy for lymph node fistula tracheobronchial tuberculosis (TBTB) in infants.

**Patients and methods:**

A retrospective analysis was undertaken on seven patients with lymph node fistula tracheobronchial tuberculosis who underwent fiberoptic bronchoscopy (FB) interventional therapy in the respiratory department of Tianjin Children's Hospital from July 2012 to July 2020. The efficacy, safety, and prognosis of CO_2_ cryotherapy intervention for the treatment of lymph node fistula TBTB in infants were summarized and analyzed.

**Results:**

Seven patients with lymph node fistula TBTB were included in this study. Their ages ranged from 6–13 months. The course of the disease from onset to TBTB ranged from 20 to 70 days. The pathological diagnoses of seven cases by FB combined with tissue biopsy were lymph node fistula TBTB, of which 28.57% (two cases) were in the early stage of rupture and 71.43% (five cases) were in the rupture stage. All patients were treated with CO_2_ cryotherapy combined with foreign body forceps and local injection drugs based on systemic antituberculosis chemotherapy. Two patients were treated once with CO_2_ cryotherapy, and five were treated three times. According to the comparison of the clinical symptoms, imaging results, and endoscopic presentations before and after the intervention, six patients achieved clinical cure, and one achieved clinical improvement. No severe intraoperative or postoperative complications were observed. The clinical symptoms, endoscopic findings, radiological manifestations, and quality of life of all patients showed marked improvement. No recurrence occurred after 3–6 months of follow-up with FB.

**Conclusion:**

CO_2_ cryotherapy can improve the treatment effect of lymph node fistula in infants with TBTB and reduce the incidence of complications. This treatment is safe and reliable in infants.

## Introduction

Tuberculosis (TB) is a disease with significant health implications and an economic burden on humans; approximately 50% of cases of TB in childhood are reported in children younger than 5 years of age (1). The incidence of primary pulmonary TB (PTB) is estimated to be 46% in children with tuberculosis (2). Tracheobronchial TB (TBTB) is the most common complication of PTB in children, accounting for 41.7–62.96% of PTB cases (3–5). TBTB is defined as microbiological and histopathological evidence of *Mycobacterium tuberculosis* in the trachea and bronchi with or without parenchymal lung lesions (6). The clinical manifestation, pathogenesis, and pathologic features of TBTB in children and adults differ owing to immunological differences (7). In adults, the active caseous necrotic form (ulcerative-necrotic type) (43.0%) is the most common type of TBTB (8). In children under 5 years of age, the neoplastic (lymph node fistula) form accounts for 73.4–96.43% (9, 10). The adjacent mediastinal or hilar lymph nodes erode the tracheobronchial wall and form bronchial lymph node fistulas in TBTB patients. Lymph node fistulas tend to compress and obstruct tracheobronchial tubes, resulting in the narrowing of the tracheobronchial lumen and inadequate secretion drainage. Ultimately, it leads to serious complications such as obstructive pneumonia, atelectasis, and lung destruction, which affect lung function and development. Due to the lack of specificity in its clinical presentation and imaging, TBTB is more likely to be misdiagnosed and underestimated in infants than in older children or adults.

The treatment of TBTB is complicated, as antituberculosis drugs have difficulty penetrating the tracheobronchial lesions. Lymph node fistula TBTB often leads to sequelae such as bronchial stenosis, although treatment with systemic antituberculosis drugs occurs without bronchoscopic intervention (8). Therefore, early bronchoscopic intervention is essential in patients with TBTB. Current interventional treatment modalities for lymph node fistula TBTB include laser therapy, cryotherapy, foreign body forceps, and local injection drugs (11). Cryotherapy has been shown to generally not damage airway cartilage, cause little to no airway perforation, have a low rate of granulation and fibrous scarring after treatment, and have fewer complications than thermal ablation (12). Consequently, cryotherapy may be more clinically helpful than interventional procedures such as thermal ablation for infants with TBTB.

There are few studies on cryotherapy for lymph node fistula TBTB in infants. This study aimed to investigate the efficacy and safety of cryotherapy for lymph node fistula TBTB in infants.

## Subjects and methods

### Study population

Seven patients aged ≤13 months who were diagnosed with lymph node fistula TBTB based on etiology, pathology, imaging, and FB results at the Tianjin Children's Hospital between July 2012 and July 2020 were included in this retrospective study. The clinical data of the participants were recorded in detail. The patients' guardians provided written informed consent for the FB, anesthesia, and their understanding of the risks of the treatment before the execution of the treatment. This study was approved by the Ethics Committee of Tianjin Children's Hospital, and the principles of the Declaration of Helsinki were followed.

### Preparation of the cryotherapy procedure

All patients received standard systemic chemotherapy for tuberculosis for 1–2 weeks before the fiberoptic bronchoscopic cryo-interventions. Computed tomography (CT), electrocardiogram, echocardiography, coagulation, hepatitis virus, syphilis, and HIV testing were performed before the procedure. Common drugs such as atropine, midazolam, adrenaline, ice/warm normal saline, and isoniazid injection were administered before treatment.

Local anesthesia with complex sedation was administered to all patients. Atropine 0.01–0.02 mg/kg was injected intramuscularly 30 min before the procedure. Lidocaine (2%) was used for nebulized inhalation and midazolam 0.2–0.3 mg/kg was injected intravenously before the procedure. During the course of the FB operation, 2% lidocaine was used as a laryngopharyngeal spray for anesthesia. The children's vital signs were monitored throughout the procedure, and the endoscopy room was equipped with first-aid equipment and medications.

### Cryotherapy instructions

CO_2_ cryo-interventions were performed using a Kulan cryo instrument (K320, China). The outer diameter of the insertion section of the bronchoscope was 4.0 mm. The operating channel was 2.0 mm, and the cryogenic probes were 1.5 mm. Cryotherapy uses extremely low temperatures to freeze local lesion tissue and destroy or remove granulomas and caseous substances in a controlled manner. When the temperature decreases below 0°C, the tissue rapidly freezes. Ice crystals form in the intracellular and extracellular tissue fluid, destroying cell structure, dehydration, denaturation of membrane system lipoproteins, and subsequent ischemic necrosis of the tissue. The frozen portion of the treatment was placed in contact with or punctured by granulomas, caseous material, or locally necrotic tissue.

### Cryotherapy procedure

FB cryo-intervention was performed under cardiac monitoring and oxygenation after anesthesia. During the procedure, a fiberoptic bronchoscope was inserted after the onset of anesthesia, and the epiglottis, larynx, trachea, healthy bronchus, and affected bronchus were examined sequentially. After TBTB lesions were found, the freezing was initiated. The temperature was maintained at −80– −70°C, and the freezing time was 15–30 s each time. The next freeze-rewarming cycle was started after rewarming. After 2–3 cycles of freezing and thawing treatments in the same region, the probe was moved around 5–6 mm, the above procedures were repeated until all lesions were frozen, and then the frozen probe was removed. After cryotherapy, the scar material and granulation tissue that obscured the lumen were removed using foreign body forceps. Finally, an isoniazid injection (50–100 mg) was administered to the lesion. Interventional therapy was performed based on routine antituberculosis chemotherapy.

### Complications and treatment

If a patient had minimal intraoperative bleeding in the bronchial mucosa, local administration of 1:10,000 epinephrine or cold saline was used to stop the bleeding. If pneumothorax or mediastinal emphysema occurred, chest imaging was performed immediately, and appropriate treatment was initiated. In the event of airway spasm, treatment with epinephrine and glucocorticoids was administered immediately until the bronchospasm was effectively controlled. In severe cases of lumen edema, oxygen was administered immediately, and antihistamines or intravenous glucocorticoids were administered. Cryotherapy is prohibited in cases of severe airway stenosis. If intraoperative hemorrhage occurred, tracheal intubation was performed, intravenous fluids and blood were administered to resolve hemorrhagic shock, and posterior pituitary hormones were also administered.

### Outcome evaluation

We collected clinical data before and after the treatment. Cough symptoms, imaging manifestations, and endoscopic manifestations were recorded and analyzed. Clinical cure was defined as the absence of cough, endoscopic demonstration of fistula healing, complete clearance of the lesion, smooth mucosa, and absence of significant narrowing of the canal lumen. CT was used to determine if the bronchial stenosis, bronchial dilatation, and pulmonary atelectasis disappeared and if more than 50% of the lung lesion was absorbed. Clinical improvement was defined as patients with occasional cough, unaffected daily sleep and life activities, endoscopic demonstration of a largely healed fistula with a reduced number or volume of lesions, mild mucosal congestion and edema, and a slight narrowing of the lumen. Clinical failure was defined as patients with continuous and frequent coughing, severe disruption of daily sleep and life activities, no closure of the fistula under endoscopy, only partial absorption or no significant change of the bronchial lesion, significantly narrowed or obstructed lumens, and no improvement or even worsening of the lung lesion on review imaging (13, 14).

### Follow-up

Follow-up included observation of worsening respiratory symptoms using bronchoscopy for endoscopic visualization. In general, follow-up evaluations were conducted once a week for the first month and then once a month. Follow-up was started 3 months after the first intervention and continued for 3–6 months. Respiratory symptoms and endoscopic visualization data were recorded and analyzed.

### Data analysis

The data were processed using SPSS 26.0. Continuous variables were expressed as median values (interquartile range). Categorical variables were expressed as percentages (%).

## Results

Seven patients were included in this study (Table 1). The median age of the seven infants was 8 months (ranging between 6–13 months). The most common symptoms at presentation were cough and sputum production. Moreover, five patients had lost weight and three had a fever. Two patients had wheezing and dyspnea. The most common comorbidity was pulmonary tuberculosis (five cases). Four (57.14%) patients had a Bacillus Calmette–Guerin scar and six (85.71%) had a history of household contact with PTB. Two patients were positive for pure protein derivative, and three patients had positive T-cell spot test results. The etiology diagnosis with a high positive rate was bronchoalveolar lavage fluid tuberculosis deoxyribonucleic acid. All patients were diagnosed with lymph node fistula TBTB *via* their endobronchial biopsy histopathological examination and macroscopic presentation results. Two cases were in the early stage of rupture and five cases were in the rupture stage; the CT findings of the patients are presented in Table 2.

**Table 1:** Demographic characteristics of the patients.

**Demographics**	***n***	**%**
**Gender**		
Male	6	85.71%
Female	1	14.29%
**Age (months)**		
≤6	2	28.57%
7–12	4	57.14%
>12	1	14.29%
**Clinical data**		
Symptoms		
Cough	7	100%
Sputum	7	100%
Wheezing	2	28.57%
Dyspnea	2	28.57%
Fever	3	42.86%
Loss of weight	5	71.43%
Co-morbidities		
Pulmonary tuberculosis	5	71.43%
Tuberculous pleurisy	1	14.29%
Tuberculous meningitis	1	14.29%
Household contact with active TB	6	85.71%
BCG vaccine, vaccination history	7	100%
Positive for BCG scar	4	57.14%
**Diagnosis**		
Immunologic diagnosis		
PPD	2	28.57%
T-SPOT	3	42.86%
Etiology diagnosis		
Sputum		
Positive AFB microscopy	0	0
Positive MTB culture	2	28.57%
BALF		
Positive in TB-DNA	3	42.86%
Positive in Gene-Xpert	2	28.57%
Endobronchial biopsy HPE		
Caseating granulomatous inflammation	7	100%
Histopathologic typing under bronchoscope		
Lymph node fistula type	7	100%
Staging of lymph node fistula TBTB		
Prophase breakdown	2	28.57%
Break-up period	5	71.43%

PPD, Pure protein derivative; T-SPOT, T-cell spot test; AFB, Acid fast bacilli; MTB, Mycobacterium tuberculosis; BALF, Bronchoalveolar lavage fluid; TB-DNA, tuberculosis deoxyribonucleic acid; HPE, Histopathological examination.

**Table 2:** Computed tomography findings of the patients before treatment.

**Computed tomography findings**	***n***	**%**
Pulmonary hilar with mediastinal lymph node enlargement	6	85.71%
Bronchial stenosis	4	57.14%
Pulmonary emphysema	2	28.57%
Pulmonary atelectasis	1	14.29%
Pleural effusion	1	14.29%
Patchy shadows	6	85.71%

**Figure 1:**
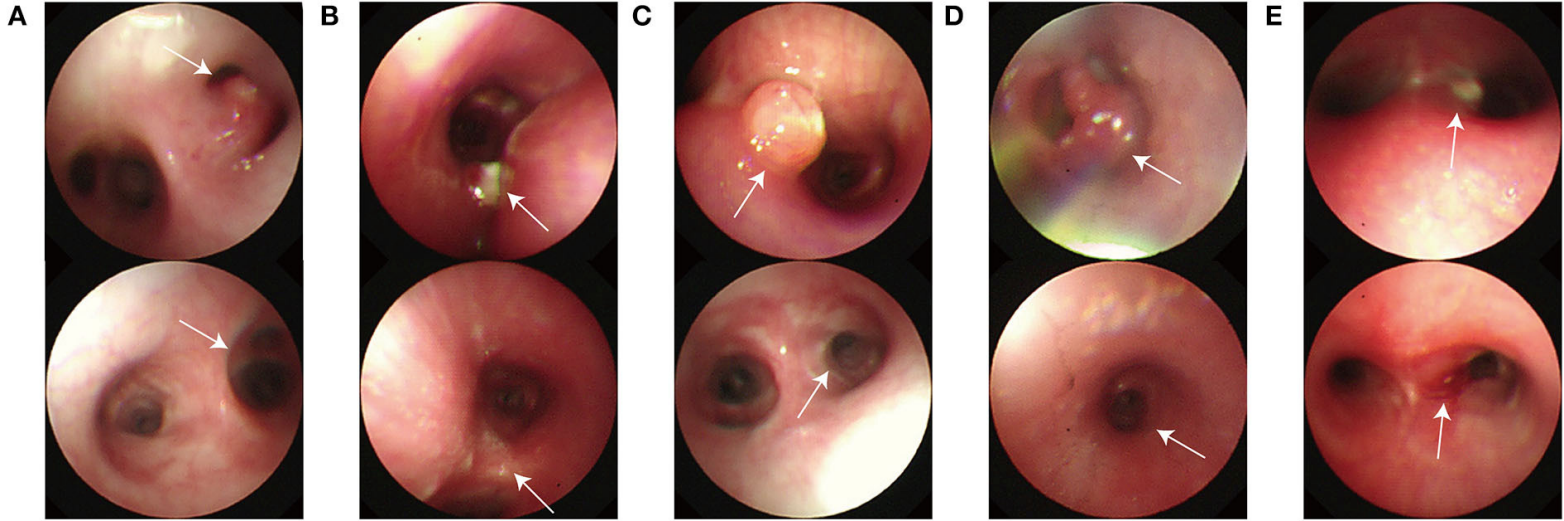
Bronchoscopic manifestations before interventional therapy and after 3 months of the first interventional therapy. **(A)** The pre-intervention endoscopic presentation that localized tracheobronchial mucosa redness, hypertrophy, hyperplasia obvious and external pressure bulge without ulceration. **(B)** The pre-intervention endoscopic presentation that localized tracheobronchial mucosa redness, hypertrophy, hyperplasia obvious and external pressure bulge with caseous substance attachment. **(C)** The pre-intervention endoscopic presentation that granulation tissue with caseous material. **(D)** The pre-intervention endoscopic presentation that granulation tissue. **(E)** The pre-intervention endoscopic presentation that the caseous material overflow (top: before interventional therapy; bottom: after interventional therapy).

Cryotherapy procedures were performed a total of 17 times; it was performed only once in two cases and three times in five patients. Bronchial involvement was seen in the right bronchus in five (71.43%) patients and the left bronchus in two (28.57%). The disease was multifocal in three cases and more commonly affected the right middle lobe. The endoscopic findings of the patients are shown in Table 3. The main type of lesion was a caseous lesion with granulation tissue. Four patients had more than one lesion type. The bronchoscopy characteristics of the patients are shown in Figure 1.

**Table 3:** Bronchoscopic lesion characteristics.

**Bronchoscopic features**	***n***	**%**
**Specific performance**		
External compression	1	14.29%
External compression with caseous	1	14.29%
Granulation tissue with caseous	3	42.86%
Simple caseous	1	14.29%
Simple granulation tissue	1	14.29%
**Lesions localization**		
Right main bronchus	1	14.29%
Right main bronchus and RML	1	14.29%
RML and RLL	2	28.57%
RLL	1	14.29%
Left main bronchus	2	28.57%
**Number of lesions**		
Right main bronchus	1	9.1%
RML	5	45.45%
RLL	3	27.27%
Left main bronchus	2	18.18%
**Interventional treatment methods**		
Cryotherapy combined with foreign body forceps and local injection drugs	7	100%
**Number of interventions**		
1 time	2	28.57%
3 times	5	71.43%

RML, Right middle lobe; RLL, Right lower lobe.

Seven patients with lymph node fistula TBTB were treated with cryotherapy combined with foreign body forceps and local injection drugs by FB. Six patients completed the full treatment course and achieved a clinical cure. One patient exhibited clinical improvement after the first intervention. However, the patient was lost to follow-up. No clinical failures occurred during the full treatment course. The mean duration from the start of therapy to the complete disappearance of the lesion was ~3 months.

Before treatment, the patients had varying degrees of cough, dyspnea symptoms, complex CT abnormalities, and endoscopic lesion features. Seven patients had continuous and frequent coughing that affected their sleep and daily life to varying degrees. Two patients had dyspnea. All patients had abnormal CT findings: 85.71% (six cases) had pulmonary hilar with mediastinal lymph node enlargement; 57.14% (four cases) had bronchial stenosis; 85.71% (six cases) had patchy shadows, and 28.57% (two cases) had pulmonary emphysema. One patient developed pulmonary atelectasis and pleural effusion. In terms of bronchoscopic lesion characteristics, the fibrobronchoscopic lesion presentations were divided into five categories: 42.86% (three cases) showed localized mucosal erythema and polyps with caseous necrotic material adherence (Figure 1C). In one case, the surface of the local tracheobronchial mucosa was red, swollen, and hypertrophic, showing an external pressure bulge without ulceration (Figure 1A). One case showed localized tracheobronchial mucosal surface erythema, hypertrophy, and an external pressure bulge with caseous material attachment (Figure 1B). One case showed granulation tissue (Figure 1D), and another showed an overflow of caseous material (Figure 1E).

**Figure 2:**
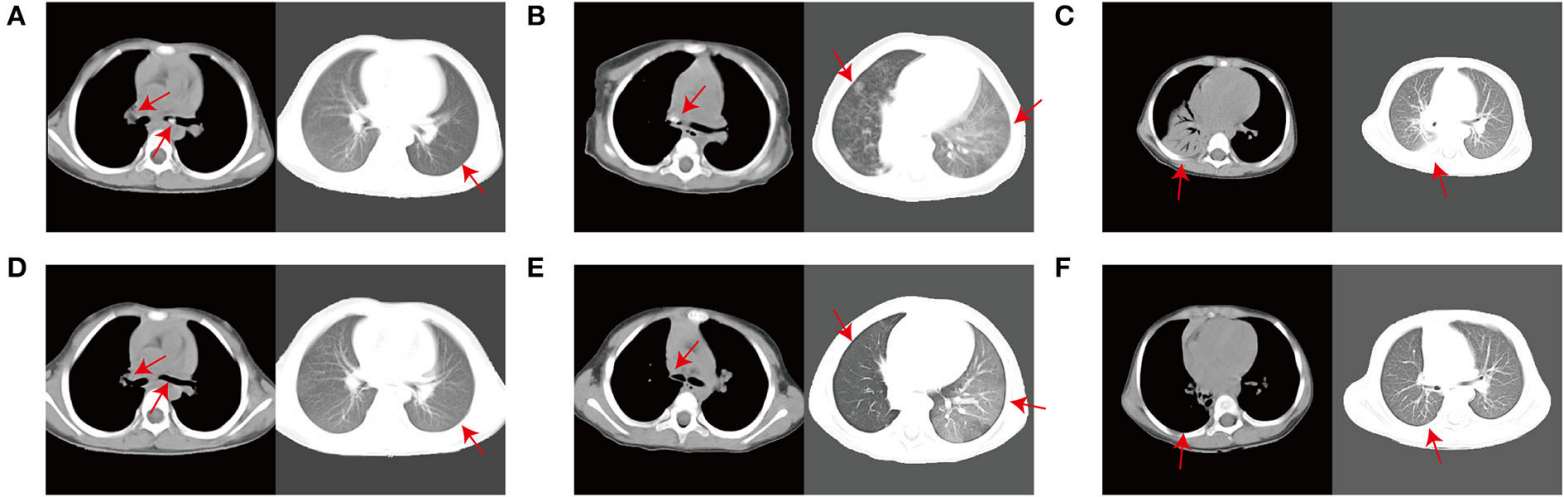
CT scan findings before interventional therapy and after 3 months of the first interventional therapy. **(A)** Pulmonary CT results of case 1 suggested a soft tissue density nodule with a dense shadow in the left main bronchus (before treatment). **(D)** CT scan results after intervention in case 1. **(B)** Pulmonary CT results of case 2 suggested mediastinal lymph node enlargement with calcification, right middle segment bronchial obstruction and scattered solid lesions in both lungs with corn-like nodular shadow (before treatment). **(E)** CT scan results after intervention in case 2. **(C)** Pulmonary CT results of case 5 suggested inflammatory solid lesions in the right lung with calcification and enlarged mediastinal lymph nodes with calcification (before treatment). **(F)** CT scan results after intervention in case 5.

After the first procedure, cough symptoms improved in all patients and disappeared in two patients with dyspnea. All patients exhibited significant improvement in lumen patency on endoscopic view. One patient was lost to follow-up after the first procedure. The remaining patients had improved cough relief, mostly healed fistulas with fewer or smaller lesions, mild mucosal congestion and edema, and a slight narrowing of the lumen. Furthermore, their CT scan revealed that their lung lesion had been absorbed more than 50%. At the end of treatment, six patients attained clinical cure in terms of their cough level (no cough), endoscopic demonstrations, and CT results following the complete treatment process. CT and FB were re-performed at the end of the interventional treatment. Compared to before the intervention, all patients showed significant improvements on lung CT in terms of hilar and mediastinal enlargement, calcified lymph nodes, bronchial obstruction, emphysema, and pulmonary atelectasis (Figure 2). The FB results in the patients showed that their fistulas were closed, their bronchial lesions were significantly absorbed and improved, their lumen stenosis or obstructions were reduced, and their bronchial mucosae were relatively smooth. A comparison of the patients' endoscopic performance is shown in Figure 1. During treatment, no significant complications occurred such as respiratory distress, hypoxemia, unexpected bleeding, pneumothorax, mediastinal emphysema, airway spasms, ductal edema, or anesthesia-related complications. A few patients had minimal bleeding when the scar material and granulation tissue that obscured the lumen were removed using foreign body forceps. Hemostasis was easily achieved by administering a small amount of epinephrine (1:10 000). The CO_2_ cryotherapy time was 10–20 min.

Six patients underwent bronchoscopic follow-up after 3 months. The bronchial lumen remained fully patent without stenosis, granulation, or scar formation in all patients, revealing a satisfactory lasting treatment effect.

## Discussion

In this study, seven infants with TBTB were treated with CO_2_ cryotherapy combined with the use of foreign body forceps and local injection drugs based on systemic antituberculosis chemotherapy. There were no obvious adverse reactions or complications during or after the interventional treatment. The clinical manifestation, imaging, and endoscopic presentation results improved significantly after the cryotherapy, and there were no cases of recurrence during the follow-up process, indicating the efficacy and safety of CO_2_ cryotherapy.

Liu et al. (10) studied 130 children (69 infants) with lymph node fistula TBTB who treated with bronchoscopic interventions; however, their study did not use cryotherapy techniques. Zhang et al. (13) studied eight patients with lymph node fistula TBTB who were treated with bronchoscopic cryotherapy; however, there were no infants in their study. In our study, lymph node fistula TBTB was reported in seven infants, and the outcome of cryotherapy on infants was studied in detail for the first time. Incidentally, the occurrence of TBTB with lymph node fistulas in children in studies outside China has been 33–38% (3, 15). Possible reasons for this are that the severity of primary TB varies between countries, regions, and hospitals.

When lymph node fistulas TBTB occur, they can significantly affect individuals' quality of life and even become life-threatening in infants owing to the special pathogenesis of TBTB, which is mainly due to the passage of pathogenic mycobacteria through the lymphatic system, resulting in local lymph node tuberculosis. Enlarged lymph nodes tend to compress the local tracheal and bronchial mucosa and submucosa, leading to local ischemia and necrosis, which predispose patients to tracheal and bronchial stenosis or complete occlusion, resulting in obstructive pneumonia, atelectasis, and pulmonary destruction (9). In this study, six patients had obstructive pneumonia, two had emphysema, and one had atelectasis and pleural effusion when they were admitted to our hospital.

Bronchoscopy plays an important role in the diagnosis of TBTB, especially in patients with suspected negative sputum smears and endobronchial diseases (16, 17). In this study, six patients had a history of household contact with TB and five patients had a disease duration of more than 1 month. The sputum smear results were negative in all patients, and the sputum culture results were negative in five patients. All patients were misdiagnosed with pneumonia before admission. The infants were diagnosed with tracheobronchial tuberculosis (lymph node fistula type) *via* their bronchoscopic tissue biopsy and macroscopic endoscopic lesion results after admission. Therefore, bronchoscopy not only improved their diagnosis of TBTB but also allowed for the determination of their type of TBTB.

The typical macroscopic bronchoscopic manifestations in children with TBTB and lymph node fistulae include caseous materials breaking into their airways. In our study, the endoscopic macroscopic manifestations in the infants were predominantly caseous granulation tissue, which is consistent with the typical features of lymph node fistula in TBTB. Moreover, in our patients, the caseous materials were accompanied by congestion, granulation tissue proliferation, or were completely encased by granulation tissue. The patients' lumens were also obstructively narrowed at the lesion sites (10).

With the development of interventional bronchoscopic techniques, several new methods, including laser, cryotherapy, foreign body forceps, and local injection drugs, in combination with systemic antituberculosis drugs, have been used to treat lymph node fistula TBTB due to their effects on accelerated lesion healing and improved prognosis (11, 18). Studies have shown that as laser damage may lead to more granulation tissue formation, laser treatment can be followed by cryotherapy to prevent granulation regeneration and maintain the lumen diameter (19). Cryotherapy can promote cicatricial fibroblast differentiation into normal fibroblasts to reduce scar and granulation tissue hyperplasia, in contrast to the effects of thermal ablation (12). In their study, Liu et al. (15) used bronchoscopic foreign body forceps and local injection drug interventions alone to treat lymph node fistula TBTB in children and reported a lower efficacy rate and more frequent interventions compared to cryotherapy.

Ni et al. (12) and Zhang et al. (13) confirmed the safety and efficacy of cryotherapy for TBTB with lymph node fistula in children. However, these studies did not directly demonstrate the applicability of cryotherapy in infants. This is an important study on CO_2_ cryotherapy interventions for lymph node fistula TBTB in infants. In this study, the signs and symptoms immediately improved following cryotherapy in all patients. Except for one patient who only achieved clinical improvement due to missed visits after the first cryotherapy session, six patients achieved clinical cure after cryotherapy. No serious complications occurred during or after the procedure, and no lesion recurred after 3–6 months of follow-up.

This study had several limitations. First, the small sample size may affect the power of our conclusions. Second, we did not include a control group without cryotherapy. Third, as all patients were enrolled from a single hospital, this might have led to biased conclusions. In future studies, it would be worthwhile to increase the sample size and further explore cryotherapy for lymph node fistula TBTB in infants. Moreover, a future multicenter study with a control group would increase the significance of the study.

## Conclusion

Bronchoscopic CO_2_ cryotherapy for lymph node fistula TBTB in infants can be performed safely and effectively in combination with the foreign body clamp method. This method can provide relief from symptoms, accelerate fistula closure, and avoid serious complications. Furthermore, the results of this study are important given that it provides pediatricians with more options and information when choosing the best treatment for infants with lymph node fistula TBTB.

## Data availability statement

The original contributions presented in the study are included in the article/supplementary material, further inquiries can be directed to the corresponding authors.

## Ethics statement

The studies involving human participants were reviewed and approved by Ethics Committee of the Tianjin Children's Hospital (No. 2021-KY-06). Written informed consent to participate in this study was provided by the participants' legal guardian/next of kin. Written informed consent was obtained from the individual(s), and minor(s)' legal guardian/next of kin, for the publication of any potentially identifiable images or data included in this article.

## Author contributions

YZ and TZ contributed to the conception of the study. TZ and NY contributed significantly to the analysis and manuscript preparation. YZ performed the data analyses and wrote the manuscript. WG and YX helped perform the analysis with constructive discussions. All authors contributed to the article and approved the final manuscript.

## Funding

This work was supported by Tianjin Natural Science Foundation (Grant Number 21JCYBJC00460), the General Project of Tianjin Children's Hospital (Grant Number Y2020013), and the Program of Tianjin Science and Technology Talent Cultivation (Grant Number RC20020).

## Conflict of interest

The authors declare that the research was conducted in the absence of any commercial or financial relationships that could be construed as a potential conflict of interest.

## Publisher's note

All claims expressed in this article are solely those of the authors and do not necessarily represent those of their affiliated organizations, or those of the publisher, the editors and the reviewers. Any product that may be evaluated in this article, or claim that may be made by its manufacturer, is not guaranteed or endorsed by the publisher.

## References

[1] Lamb GS Starke JR. Tuberculosis in infants and children. Microbiol Spectr. (2017) 5. 10.1128/microbiolspec.TNMI7-0037-2016 28387193 PMC11687478

[2] Wu XR Yin QQ Jiao AX Xu BP Sun L Jiao WW. Pediatric tuberculosis at Beijing children's hospital: 2002-2010. Pediatrics. (2012) 130:e1433–40. 10.1542/peds.2011-3742 23184116

[3] de Blic J Azevedo I Burren CP Le Bourgeois M Lallemand D Scheinmann P. The value of flexible bronchoscopy in childhood pulmonary tuberculosis. Chest. (1991) 100:688–92. 10.1378/chest.100.3.688 1909618

[4] Chan S Abadco DL Steiner P. Role of flexible fiberoptic bronchoscopy in the diagnosis of childhood endobronchial tuberculosis. Pediatr Infect Dis J. (1994) 13:506–9. 10.1097/00006454-199406000-00008 8078738

[5] Cakir E Uyan ZS Oktem S Karakoc F Ersu R Karadag B. Flexible bronchoscopy for diagnosis and follow up of childhood endobronchial tuberculosis. Pediatr Infect Dis J. (2008) 27:783–7. 10.1097/INF.0b013e318170fccc 18664928

[6] Hoheisel G Chan BK Chan CH Chan KS Teschler H Costabel U. Endobronchial tuberculosis: diagnostic features and therapeutic outcome. Respir Med. (1994) 88:593–7. 10.1016/S0954-6111(05)80007-1 7991884

[7] Alcaïs A Fieschi C Abel L Casanova JL. Tuberculosis in children and adults: two distinct genetic diseases. J Exp Med. (2005) 202:1617–21. 10.1084/jem.20052302 16365144 PMC2212964

[8] Chung HS Lee JH. Bronchoscopic assessment of the evolution of endobronchial tuberculosis. Chest. (2000) 117:385–92. 10.1378/chest.117.2.385 10669679

[9] Jiao AX Sun L Liu F Rao XC Ma YY Liu XC. Characteristics and clinical role of bronchoscopy in diagnosis of childhood endobronchial tuberculosis. World J Pediatr. (2017) 13:599–603. 10.1007/s12519-017-0046-1 28623556

[10] Liu F Rao XC Ma YY Meng CF Pan YN Liu H. Classification of tracheobronchial tuberculosis in 252 children. Chin J Tuberc Res Dis. (2022) 45:282–8. 10.3760/cma.j.cn112147-20210624-00444 35279992

[11] Sriratanaviriyakul N Lam F Morrissey BM Stollenwerk N Schivo M Yoneda KY. Safety and clinical utility of flexible bronchoscopic cryoextraction in patients with non-neoplasm tracheobronchial obstruction: a retrospective chart review. J Bronchology Interv Pulmonol. (2015) 22:288–93. 10.1097/LBR.0000000000000203 26439016

[12] Ni C Yu H. Han X Meng C Zhang Y. Clinical analysis of bronchoscopic cryotherapy in 156 pediatric patients. Pediatr Int. (2017) 59:62–7. 10.1111/ped.13088 27396528

[13] Zhang HS Chen XP Ye LP Wang GF Zheng YM Zhang HL. Clinical application of transbronchial cryotherapy in the diagnosis and treatment of tracheobronchial tuberculosis in childre*n*. Chin J Pediatrics. (2021) 59 963–7. 10.3760/cma.j.cn112140-20210504-00378 34711032

[14] Schmidt M Thomsen M Schmidt U. Suitability of ivy extract for the treatment of paediatric cough. Phytother Res. (2012) 26:1942–7. 10.1002/ptr.4671 22532491

[15] Liu F Rao XC Ma YY Meng CF Pan YN Jiao AX. Evaluation of the effectiveness of transbronchoscopic intervention for tracheobronchial tuberculosis in children with lymph node fistula. Chin J Anti-Tuberc. (2021) 43:826–31. 10.3969/j.issn.1000-6621.2021.08.014

[16] Mondoni M Repossi A Carlucci P Centanni S Sotgiu G. Bronchoscopic techniques in the management of patients with tuberculosis. Int J Infect Dis. (2017) 64:27–37. 10.1016/j.ijid.2017.08.008 28864395

[17] Zhang Q Zhang Q Sun BQ Liu C Su AN Wang XH. GeneXpert MTB/RIF for rapid diagnosis and rifampin resistance detection of endobronchial tuberculosis. Respirology. (2018) 23:950–5. 10.1111/resp.13316 29691960

[18] Kut A Cakir E Gokdemir Y Midyat L Ersu R Erdem E. Intrinsic endobronchial obstructions in children from Turkey: evaluation of 2,555 flexible bronchoscopic procedures. Respiration. (2013) 85:43–8. 10.1159/000342339 23006581

[19] Kashyap S Solanki A. Challenges in endobronchial tuberculosis: from diagnosis to management. Pulm Med. (2014) 2014:594806. 10.1155/2014/594806 25197570 PMC4147266

